# Analysis of parametric instability of cigarettes based on computational fluid dynamics methods

**DOI:** 10.1016/j.heliyon.2023.e19449

**Published:** 2023-08-24

**Authors:** Jiaxin Wei, Hui Xiao, Xiaoming Wang, Hang Zhao, Xiushan Wang, Sen Yao, Binqiang Tian, Wangshen Hao

**Affiliations:** a Xuchang Cigarette Factory of Henan Zhongyan Industry Co., Ltd, Xuchang, 461001, China; b College of Mechanical and Electrical Engineering, Henan Agricultural University, Zhengzhou, 450002, China; c School of Mechanical and Power Engineering, Zhengzhou University, Zhengzhou, 450002, China

**Keywords:** Cigarette ventilation rate, Calculation flow physics, Porosity, Simulation analysis, Cigarette material

## Abstract

In actual production tests, there are large fluctuations in the ventilation rate and smoking resistance of cigarettes. In this paper, fluctuations in ventilation rate and smoking resistance are attributed to variations in the number of ventilation holes in the joint paper and the porosity of each component. The effects of small changes in porosity of each component were analyzed by using computational fluid dynamics methods. The results showed that the difference in the effective number of ventilation holes caused by the gluing process had little effect on the ventilation rate and smoking resistance of cigarettes in the practical production process. The smoking resistance is mainly affected by the structural parameters of filter tow and cut tobacco section in the length direction of cigarettes, the porosity of cigarette paper and packaging paper is positively related to the cigarette ventilation rate, the porosity of cut tobacco and the porosity of filter tow are negatively related to the cigarette ventilation rate, and the influence of shaped paper is the smallest. The results can provide technical and theoretical basis for the optimization of cigarette process parameters and the stabilization of ventilation rate.

## 1 Introduction

Cigarette ventilation rate affects consumer smoking experience and product safety, and is one of the product quality evaluation indicators that cigarette companies are very concerned about [1,2]. Since there are many factors that cause the fluctuation of cigarette ventilation rate, the stable control of ventilation rate is more difficult in the actual production process.

To address this problem, many researchers have used computational fluid dynamics to simulate the distribution characteristics of the internal airflow field of cigarettes and to study the effects of cigarette materials and process parameters on cigarette smoking resistance and ventilation rate [3,4]. Baker et al. [5] applied Darcy's law to simulate the distribution of simple airflow fields in and around the combustion end face of a cigarette in a numerical simulation calculation of a cigarette. Saidi et al. [6] used numerical simulation to analyze the smoking resistance of cigarettes in the lit state. Eitzinger et al. [7] used numerical analysis to study the effect of cigarette material on the smoking resistance of cigarettes by nonlinear instanton equation and derived the nonlinear pressure drop and flow rate relationship in unlit cigarettes. Wang et al. [8] established a three-dimensional gas flow mathematical model to simulate and verify the spatial distribution pattern of pressure drop and rate in unlit 3R4F cigarettes under 17.5 ml/s constant-flow smoking condition, and simulated the relationship between pressure drop and smoking flow rate in each section of cigarettes, which provides a reference for the setting of parameters under the numerical simulation of cigarettes. Yu et al. [9] used a mathematical modeling approach to study the effect of changes in physical parameters of cigarette on CO in smoke. Wu et al. [10] used CFD simulation software to construct a 3D fluid simulation model of cigarette and the surrounding air domain, and compared the simulated values of cigarette paper intake and cigarette smoking resistance with the real values to verify the accuracy of the model, which is a guideline for setting the parameters of CFD simulation calculation. Several other researchers also analyzed and studied the factors affecting the ventilation rate of cigarette by means of mathematical statistics and experiments [[11], [12], [13]].

The existing studies provide references and bases for the numerical simulation calculation of the internal airflow field of cigarette [14,15]. However, most of the current studies establish mathematical models and describe the distribution of pressure and velocity inside the cigarette, and the influence law of the structural parameters of each cigarette component on the cigarette ventilation rate and smoking resistance is not clear.

The complete cigarette consists of five parts: acetate filter rod, filter rod forming paper, tobacco, and cigarette paper, and the joint paper connects the filter rod and cigarette parts [16]. From the microstructure, all five parts are fibrous and porous with different degrees of permeability. From the perspective of computational fluid dynamics, the parameters of tobacco production such as density, weight and proportion of tobacco are only affected by the porosity of the porous structure when the fiber composition is the same. At the same time, the gluing process in the production process will cause the difference in the number of ventilation holes in the cigarette, which will affect the flow of gas from the outside to the inside of the cigarette from the geometric structure.

Therefore, this paper attributes the fluctuations of ventilation rate and smoking resistance to the changes in the number of ventilation holes and the porosity of each component on the splicing paper. By establishing a three-dimensional flow model of a brand of cigarettes, the effects of the changes in the porosity of each component on the ventilation rate and smoking resistance of cigarettes with different numbers of ventilation holes under constant-flow smoking are investigated in an attempt to find out the key factors affecting the ventilation rate and smoking resistance of cigarettes and to provide theoretical support for the setting of cigarette process parameters.

## 2 Numerical model

### 2.1 Geometric model

**Fig. 1:**
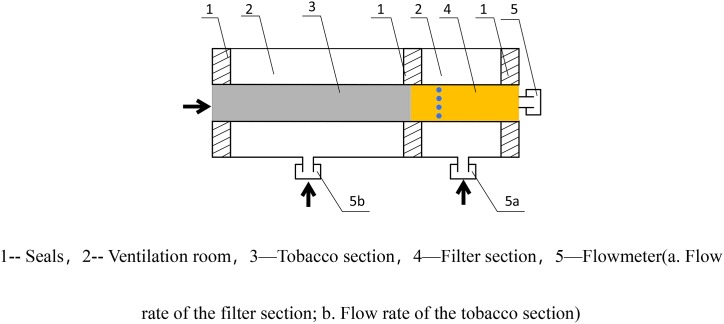
Measurement principle of cigarette ventilation rate.

The ventilation rate testing equipment is a comprehensive test bench for the physical properties of cigarettes in Xuchang Cigarette Factory of Henan Cigarette Industry Co. The platform is capable of directly measuring a number of parameters such as cigarette length, circumference, ventilation rate, and smoking resistance. Cigarette ventilation rate is measured according to GB/T 2283815–200, and the principle of cigarette ventilation rate measurement is shown in Fig. 1.

**Fig. 2:**
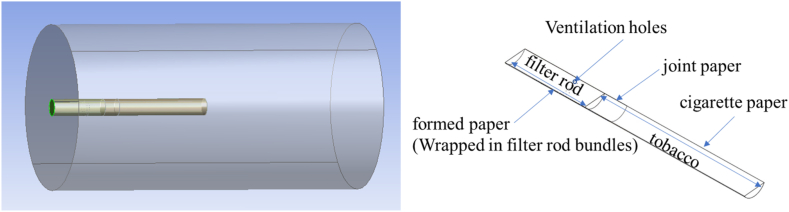
Three-dimensional model of cigarette.

The geometric model of the smoke ventilation rate testing process is established according to the actual testing situation, as shown in Fig. 2. The cigarettes to be tested were 120 cigarettes of a certain brand provided by Xuchang Cigarette Factory of Henan China Tobacco Industry Co. And the relevant parameters are shown in Table 1. In order to make the model output more realistic to the state of the cigarette during the test, a columnar air domain is designed on the outside of the cigarette model. The diameter of the air domain is 10 times the diameter of the cigarette, and the end face of one side is flush with the end face of the cigarette filter. In the model, the paper materials are all designed to have a uniform air intake surface, including cigarette paper, filter rod forming paper and joint paper. In this case, the ventilation holes on the joint paper are directly modeled with real dimensions.

**Table 1:** Parameters of cigarette physical properties.

Geometric parameters	Value	physical properties	Value
Length of cigarette paper/mm	50	Porosity of the tobacco section	0.686
Thickness of cigarette paper/μm	7.79	Porosity of the filter rob	0.858
Length of tobacco section/mm	50	Resistance characteristic of the tobacco section/m^−2^	1.21 × 10^9^ [10]
Length of filter rod/mm	25	Resistance characteristics of cigarette paper/m^−2^	1.643 × 10^14^ [10]
Diameter of filter rod/mm	7.59	Resistance characteristics of joint paper/m^−2^	3.2 × 10^14^ [10]
Length of formed paper/mm	25	Resistance characteristics of formed paper/m^−2^	9.44 × 10^12^ [10]
Thickness of formed paper/μm	20	Resistance characteristics of filter rod/m^−2^	3.98 × 10^9^ [10]
Length of joint paper/mm	32		
Thickness of joint paper/μm	45		

### 2.2 Physical model

Based on the physical characteristics of cigarette materials, the following assumptions are made in this model: (1) the filter rod bundle, cigarette bundle, cigarette paper, joint paper, and filter rod forming paper are all isotropic porous media, and the flow of fluid in the media is described by Darcy's law, i.e., the percolation rate is proportional to the pressure gradient; (2) The gas flow inside the cigarette is incompressible flow. The control equations include the mass conservation equation and the momentum conservation equation [17], as shown in Equation (1) and Equation (2).

(1) $$\frac{\partial \left(\varepsilon \rho \right)}{\partial t}+\nabla \cdot \left(\varepsilon \rho \vec{v}\right)=0$$

(2) $$\frac{\partial \left(\varepsilon \rho \vec{v}\right)}{\partial t}+\nabla \cdot \left(\varepsilon \rho \vec{v}\vec{v}\right)=-\varepsilon \nabla p+\nabla \cdot \left(\varepsilon \vec{\tau }\right)+\varepsilon {\vec{B}}_{f}-\left(\frac{\varepsilon^{2}\mu }{\alpha }\vec{v}+\frac{\varepsilon^{3}C_{2}}{2}\rho \left|\vec{v}\right|\vec{v}\right)$$

where ε is the porosity, $\vec{v}$ is the fluid velocity (m/s), $\vec{\tau }$ is the shear force, ${\vec{B}}_{f}$ is the bolumetric force, *ρ* is the density (kg/m^3^), *μ* is the aerodynamic viscosity (Pa·s), *p* is the pressure (Pa). ***α*** is the permeability and *C*_2_ is the inertial resistance coefficient, determined from Eqs. (3), (4), respectively, according to ergun's equation.

(3) $$\alpha =\frac{D_{p}^{2}}{150}\frac{\varepsilon^{3}}{{\left(1-\varepsilon \right)}^{2}}$$

(4) $$C_{2}=\frac{3.5}{D_{p}}\frac{\left(1-\varepsilon \right)}{\varepsilon^{3}}$$

where *D*_p_ is the average diameter of the porous structure. The standard parameters of each structure of the smoke branch shown in Table 1 are used to calculate the inverse problem of the average diameter with the standard parameters when the porosity changes.

The ventilation rate is calculated as shown in Equation (5).

(5) $$V=V_{c}+V_{M}=\frac{Q_{c}+Q_{M}}{Q}\times 100\%$$

where *V* is the total ventilation rate of the cigarette, *V*_c_ is the ventilation rate of the cigarette paper section, *V*_m_ is the ventilation rate of the filter section, *Q*_c_ is the air intake of the cigarette paper section, and *Q*_m_ is the air intake of the filter section.

The above control equations are solved using Ansys Fluent. The mesh was created using Fluent Meshing software and the mesh type was unstructured polyhedral mesh. The number of meshes was determined to be 1916125 by the mesh independence test. The inlet condition was set as a pressure inlet, the outlet flow rate of the cigarette filter *Q*_out_ = 17.5 ml/s, and the fluid parameters used the air properties at 25 °C measured by the temperature measurement device that comes with the instrumentation. The viscosity was calculated formally by Brinkman revision.

## 3 Results and discussion

According to GB/T 16447–2004 and GB/T 22838.15–2009 [18], 120 cigarettes of a brand to be tested provided by Xuchang Cigarette Factory of Henan China Tobacco Industry Co., Ltd. Were equilibrated at (22 ± 2)°C and relative humidity (60 ± 5)% which are read by temperature and humidity meters around the interior of the workshop for 48 h. The cigarettes were numbered and used as a backup, and then sent to the ventilation rate testing equipment (CERULEAN QTM 5UC, UK) for ventilation rate and resistance testing. After the test, the cigarettes were disassembled and the number of effective ventilation holes was counted, and the model calculation results were validated, and the validated model was subsequently used for the study.

### 3.1 Model validation

The results of all the cigarette tests were calculated and compared with the results of the model with an effective number of 38 holes. The number of effective ventilation holes was 38.53 ± 1.32, the total ventilation rate was 13.1 ± 1.52%, and the smoking resistance was 985 ± 41 Pa. The model results for the 38-hole case showed a smoking resistance of 966 Pa and a ventilation rate of 13.34%. Comparing the simulated and measured average values of cigarette ventilation rate and smoking resistance, the error between the simulated and measured average values of cigarette resistance was 19 Pa, and the error between the simulated and measured average values of cigarette ventilation rate was 0.18%. The errors of cigarette smoking resistance and ventilation rate were both small.

**Fig. 3:**
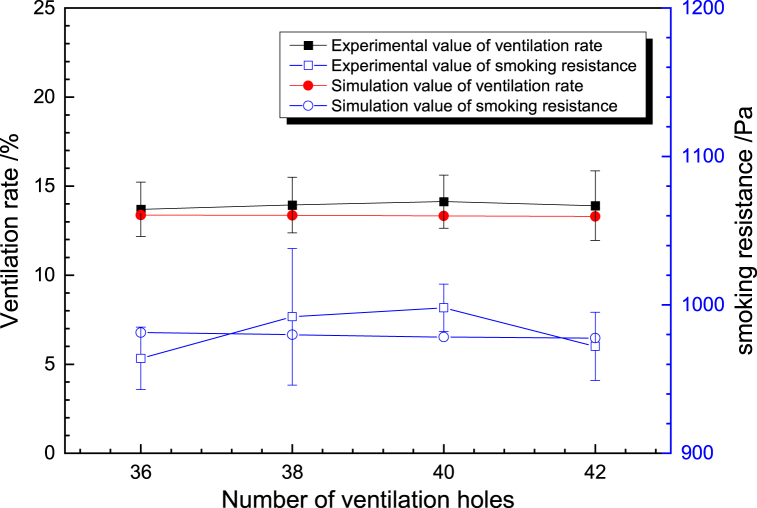
Model validation.

To further verify the reliability of the model, geometric models with different number of holes were established and compared with the experimental results, as shown in Fig. 3. The results show that the ventilation rate and the smoking resistance are in good agreement with the experimental results, and the slight difference with the average value is mainly due to the variability of the structure of each component of the cigarette due to the limitation of the process, and the overall model calculation results are more reliable.

### 3.2 Pressure and velocity distribution

**Fig. 4:**
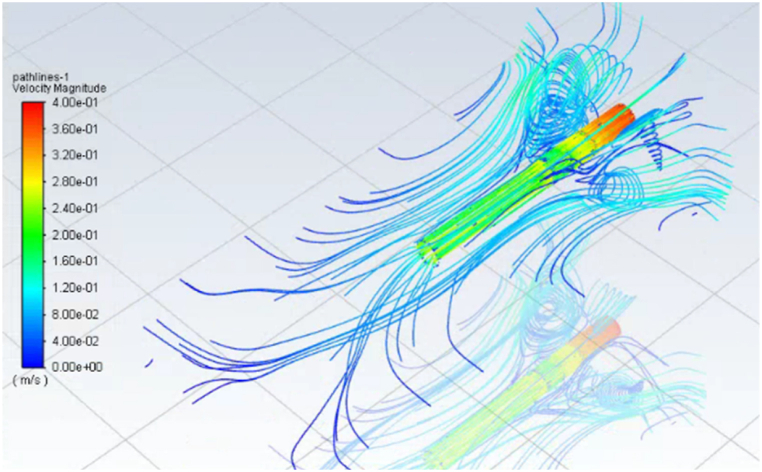
Flow field distribution diagram of cigarette.

**Fig. 5:**
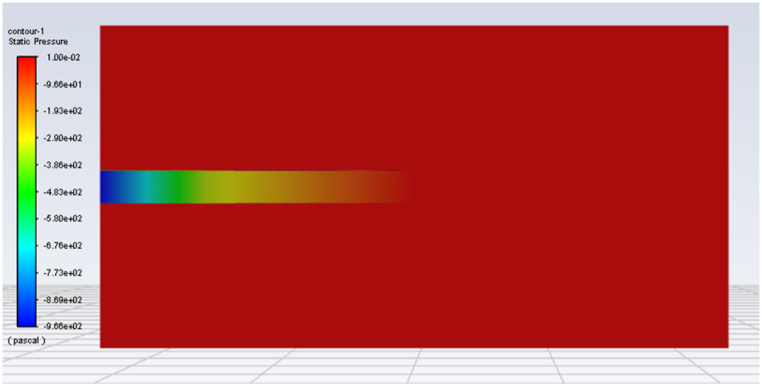
Distribution of pressure on the median plane.

Post-processed to further analyze the internal airflow field distribution characteristics and pressure variation characteristics of unlit cigarettes in constant-flow smoking mode, as shown in Fig. 4, Fig. 5.

Fig. 4 shows the flow distribution of the airflow field inside and outside of a standard cigarette under constant-flow smoking. From Fig. 4, it can be seen that under the constant-flow smoking mode of 17.5 ml/s, the air outside the cigarette mainly enters the interior of the cigarette from three sections: the end surface of the tobacco, the cigarette paper and the ventilator. The air inside the cigarette has a small change in velocity near the filament section, and after passing through the cross section of the vent hole in the filter section, the flow rate inside the cigarette suddenly increases, and the increased airflow comes from the inlet air at the vent hole. The air entering the interior of the cigarette through the cigarette paper and the vent hole accounted for 13.3% of the total air intake.

Fig. 5 shows the pressure drop distribution characteristics of a standard cigarette under constant-flow smoking. The pressure gradient is mainly concentrated at the position of the vent hole and the position of the interface between the filter rod and the tobacco, and the lowest pressure point is obtained at the exit of the filter, and the maximum pressure difference is 966 Pa. On the whole, the pressure change is mainly concentrated along the length of the cigarette, i.e., the change of the smoking resistance is mainly affected by the resistance along the length of the cigarette. The pressure variation was mainly concentrated along the length of the cigarette, i.e., the change of puff resistance was mainly influenced by the resistance along the cigarette length. Based on the pressure distribution, it can be judged that the structure of the filter rod and tobacco section have greater influence on the smoking resistance, while the forming paper, the cigarette paper and the joint paper have a much smaller thickness than the length direction, and their own porosity and permeability are much smaller than the filter rod and tobacco section, so it is inferred that they only have an influence on the ventilation rate, and the specific influence needs to be further studied.

### 3.3 The effect of porosity of each structure of cigarette on the smoking resistance and ventilation rate

**Fig. 6:**
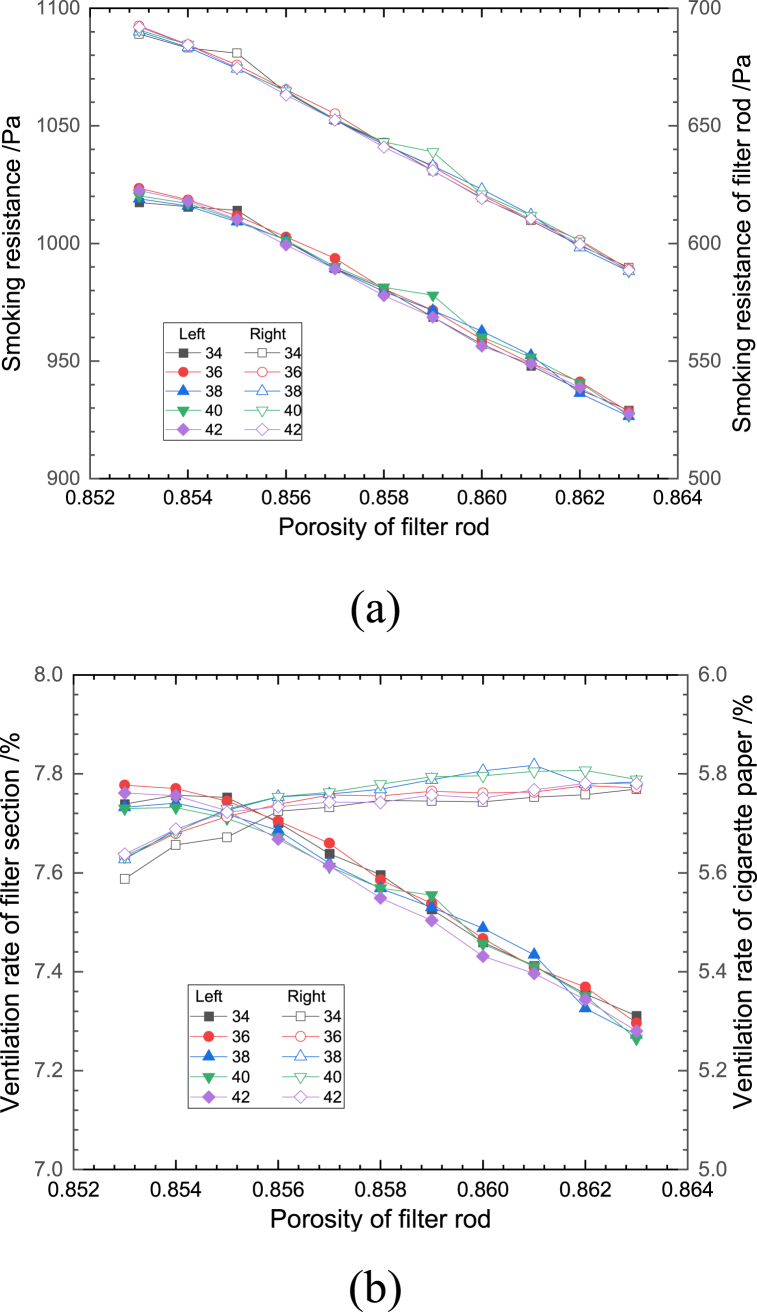
Effect of porosity of filter rod on (a) smoking resistance and (b) ventilation rate.

**Fig. 7:**
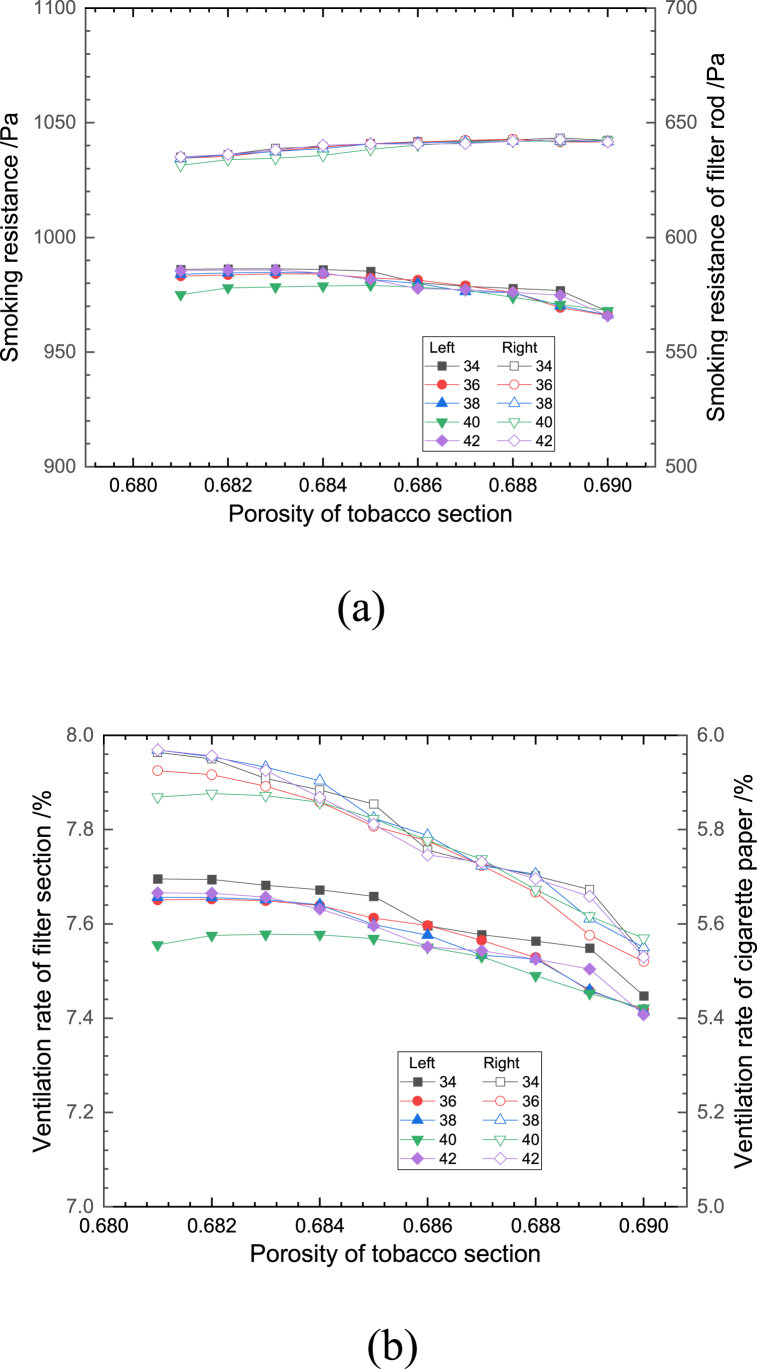
Effect of porosity of tobacco section on the (a) smoking resistance and (b) ventilation rate.

**Fig. 8:**
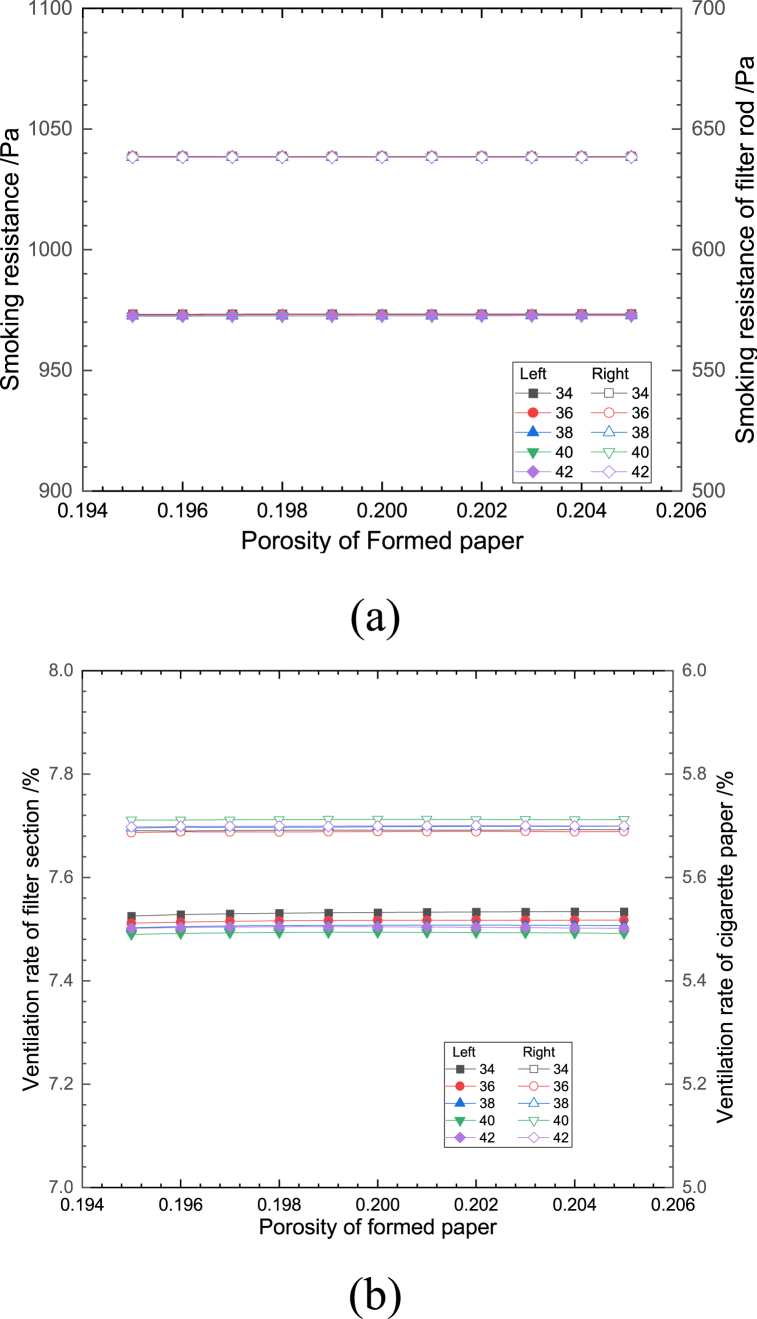
Effect of porosity of formed paper on (a) smoking resistance and (b) ventilation rate.

**Fig. 9:**
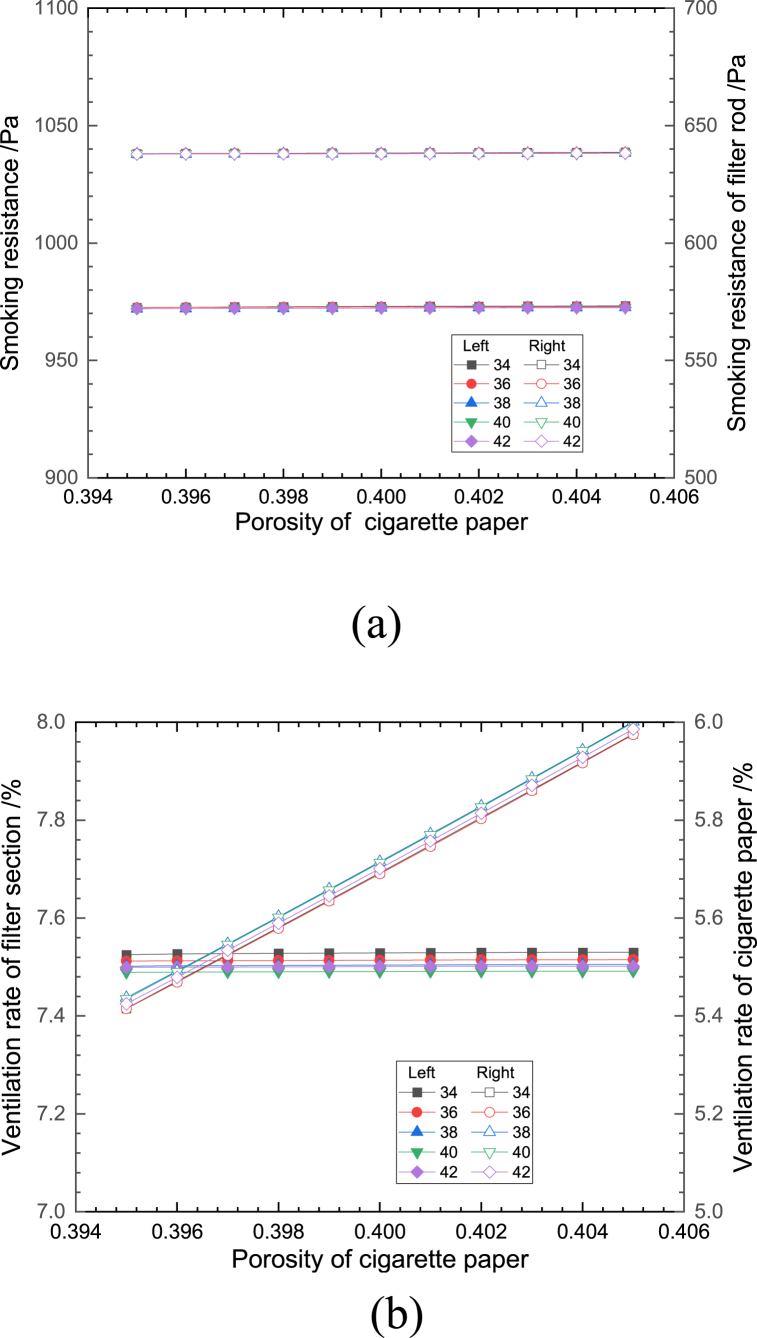
Effect of porosity of cigarette paper on (a) smoking resistance and (b) ventilation rate.

**Fig. 10:**
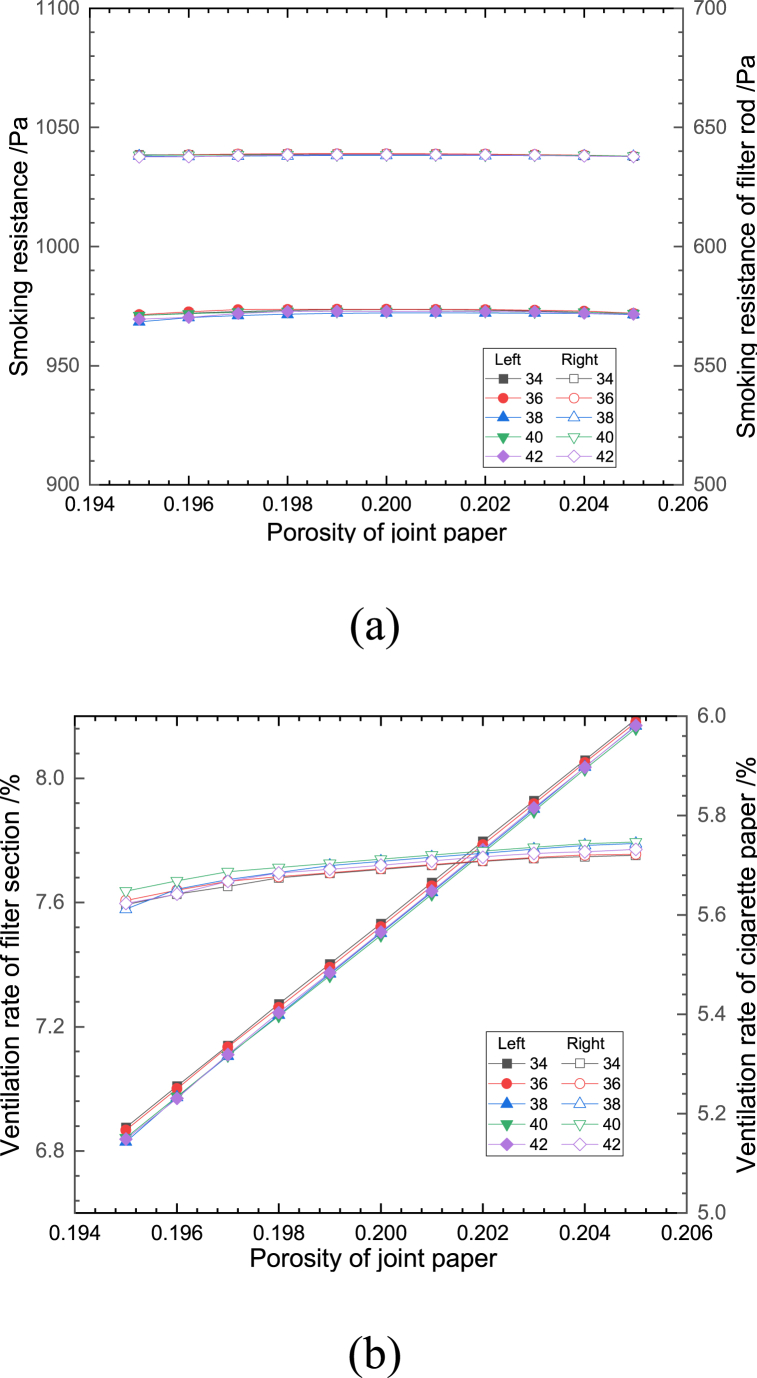
Effect of porosity of joint paper on (a) smoking resistance and (b) ventilation rate.

Considering that the production process of cigarettes is a standardized process, only the effect of small amount of variation (±0.005) on the smoking resistance and ventilation rate in the standard case is considered. Combined with the effective number of ventilation holes obtained from the experimental statistics, the effective numbers of ventilation holes were taken as 34, 36, 38, 40 and 42, and the geometric models were established and analyzed the effects of the changes of porosity of each structure of cigarettes on the ventilation rate and the smoking resistance of cigarettes, as shown in Fig. 6, Fig. 7, Fig. 8, Fig. 9, Fig. 10.

Fig. 6(a and b) and shows the effect of filter rod bundle porosity on the smoking resistance and the ventilation rate for different number of ventilation holes. As shown in Fig. 6, with the same number of ventilation holes, as the porosity of the filter rod bundle increased, the total and filter section resistance decreased, which is consistent with the universal judgment that the internal airflow resistance decreases when the porosity of the filter rod bundle increases; the total ventilation rate of cigarettes decreased by 2.31%, of which the ventilation rate of the filter section decreased by 6.44%, and the ventilation rate of the cigarette paper section increased by a relatively small amount. The growth rate was 3.62%, which was mainly due to the reduction of radial resistance in the cigarette filter section and the increase of air flow into the cigarette from the end of the tobacco and the cigarette paper. At the same porosity, the number of ventilation holes has a relatively small effect on the smoking resistance, and the smaller the number of ventilation holes, the greater the ventilation rate of the filter section and the smaller the ventilation rate of the tobacco section.

Fig. 7(a and b) shows the effect of changing the porosity of the tobacco section on the smoking resistance and the ventilation rate at different numbers of effective ventilation holes. At the same number of ventilation holes, with the increase of the filament section porosity, the overall cigarette smoking resistance showed a decreasing trend with a decrease rate of 2.03%, while the filter rod smoking resistance showed an increasing trend with an increase rate of 1.26%. This is mainly due to the fact that the decrease of flow resistance caused by the increase of porosity in the tobacco section is mainly concentrated in the tobacco section, and the average gas flow rate in the filter rod section increases because the structural characteristics of the filter rod end do not change, which in turn causes the increase of flow resistance in the filter rod section. The total ventilation rate of cigarettes showed a decreasing trend, with a decrease rate of 3.22%. This is mainly due to the fact that with the increase of the porosity of the cigarette section, the total cigarette smoking resistance decreases, and the proportion of the gas flow rate from the end face of the cigarette into the interior of the cigarette increases, resulting in the proportional decrease of the gas flow rate from the cigarette paper and ventilation holes into the interior of the cigarette, and then the ventilation rate of the cigarette paper section decreases. The ventilation rate of the filter section decreases with the increase of the number of ventilation holes, which is mainly due to the increase of the flow resistance in the vertical direction caused by the increase of the flow velocity of the filter rod in the direction of the cigarette, resulting in the decrease of the fluid entering the cigarette through the small holes.

Fig. 8(a and b) shows the effect of changing the porosity of the filter rod forming paper on the smoking resistance and the ventilation rate of cigarettes at different effective numbers of ventilation holes. It can be seen from the figure that, with the increase of the porosity of the filter rod forming paper when the number of ventilation holes remains unchanged, the changes of the ventilation rate and smoking resistance of both the filter and cigarette paper sections are less than 0.1%。The reason is that the filter rod is much smaller in the thickness direction than in the length direction, the flow inside the forming paper is mainly dominated by the fluid flowing through the ventilation holes, and because the paper structure itself has a greater resistance to flow compared to the filter rod filament bundle and the tobacco section, small changes in its porosity do not significantly affect the flow resistance of the components inside the tobacco stick. When the porosity does not change, the number of ventilation holes does not have a significant effect on the smoking resistance, and the greater the number of ventilation holes, the smaller the ventilation rate of the filter section and the greater the ventilation rate of the cigarette paper section. This is mainly because when the number of ventilation holes decreases, the pressure gradient at the ventilation holes increases, which makes the fluid flow through the ventilation holes increase.

Fig. 9(a and b) shows the effect of cigarette paper porosity change on the smoking resistance and the ventilation rate at different effective number of ventilation holes. The average growth rate of cigarette smoking resistance was less than 0.1% with the increase of cigarette paper porosity when the number of ventilation holes was the same. The total ventilation rate of cigarettes showed an increasing trend with a growth rate of 4.37%, among which the ventilation rate of the filter remained unchanged, and the ventilation rate of the cigarette paper section showed an approximately linear growth trend with a growth rate of 10.1%, because as the porosity of cigarette paper increased, more air entered through the cigarette paper and the ventilation rate of the cigarette paper section increased, leading to an increase in the total ventilation rate of cigarettes. At the same porosity, the effect of the number of ventilation holes on the smoking resistance and porosity is consistent with that of the forming paper.

Fig. 10(a and b) shows the effect of the change of joint paper porosity on the smoking resistance and the ventilation rate of cigarettes at different numbers of effective ventilation holes. When the number of ventilation holes is kept constant, the effect of the change of the jointed paper porosity on the smoking resistance is relatively small, with the increase of the porosity, the ventilation rate of the filter section increases, and the ventilation rate of the cigarette paper section increases slightly, so that the overall ventilation rate also increases. This is mainly due to the fact that the internal flow resistance of the splice paper is larger compared with that of the forming paper and cigarette paper, and it is directly connected to the external air environment. When the porosity increases, the internal flow resistance decreases, and it is easier for the fluid on the outside of the filter to enter the cigarette structure due to the proximity of the draw end, which increases the ventilation rate of the filter section. At the same time, due to the increase of pressure gradient in the direction of the cigarette branch in the filter section, the pressure at the junction of the filter rod and the tobacco section is more similar to the pressure at the end of the tobacco section, and the pressure change in the tobacco section is mainly concentrated in the side near the filter rod, which makes the fluid entering into the interior of the cigarette branch through the end of the tobacco relatively less, and the ventilation rate of the cigarette section slightly increases.

In summary, the differences in the trends of the fold lines corresponding to different effective numbers of ventilation holes in each of the graphs shown in Fig. 6, Fig. 7, Fig. 8, Fig. 9, Fig. 10 are small, with an average deviation of less than 0.1%, indicating that a small change in the effective number of ventilation holes does not have a significant effect on the ventilation rate and smoking resistance of cigarettes. The fluctuations in smoking resistance and ventilation rates that occur in production are mainly influenced by changes in porosity caused by a variety of factors. In terms of the smoking resistance, it can be seen from Fig. 6, Fig. 7 that the variation of structural parameters in the length direction of the cigarette is the main reason for the variation of the smoking resistance, and the variation of parameters of the three paper structures has minimal effect on the smoking resistance, which is consistent with the results of the previous analysis. The relationship between the ventilation rate and the structural parameters of each component is more complicated, except for the filter rod forming paper, which is located between the splice paper and the filter rod filament bundle, the change of porosity will affect the ventilation rate to a larger extent.

## 4 Conclusions

In this paper, a three-dimensional fluid simulation model of a brand of cigarettes from Xuchang Cigarette Factory of Henan Cigarette Industry Co., Ltd. Was established by the method of computational fluid dynamics, and the effects of structural parameters of cigarette components on the smoking resistance and ventilation rate were studied. The following conclusions were obtained.

(1) During the practical production of cigarettes, the difference in the effective number of ventilation holes caused by the gluing process has a small effect on the ventilation rate and smoking resistance of cigarettes.
(2) The smoking resistance was mainly influenced by the structural parameters of the filter rod bundle and the tobacco section in the length direction of the cigarette, and the changes of the parameters of the three paper structures of the forming paper, cigarette paper and joint paper had less influence on the smoking resistance. With the increase of the porosity of the tobacco section and the porosity of the filter rod bundle, the cigarette smoking resistance showed a decreasing trend, and the decrease rates were 2.03% and 9.80%, respectively.
(3) The relationship between the ventilation rate and the structural parameters of each component is complex. Except for the forming paper, the variation of the porosity of the other four structures will affect the ventilation rate to a large extent. Cigarette paper porosity and jointing paper are positively correlated with cigarette ventilation rate; the porosity of the tobacco part and the porosity of the filter rod bundle are negatively correlated with cigarette ventilation rate.
(4) Although our calculations are based on specific types of short cigarettes, the findings are also relevant for other types of cigarettes. This study can provide theoretical guidance for revealing the law of airflow movement in cigarette smoking process and optimizing the design parameters of cigarette process.

## Author contribution statement

JiaXin Wei: Conceived and designed the experiments; Wrote the paper. Hui Xiao; XiaoMing Wang: Analyzed and interpreted the data. Hang Zhao: Performed the experiments; Analyzed and interpreted the data; Wrote the paper. XiuShan Wang: Conceived and designed the experiments; Contributed reagents, materials, analysis tools or data. Sen Yao: Conceived and designed the experiments; Performed the experiments; Wrote the paper. BinQiang Tian; WangShen Hao: Contributed reagents, materials, analysis tools or data.

## Data availability statement

Data will be made available on request.

## Declaration of competing interest

The authors declare that they have no known competing financial interests or personal relationships that could have appeared to influence the work reported in this paper
